# Role of viral and host factors in determining the outcome of HBV-associated acute liver failure

**DOI:** 10.1186/s12967-026-08347-z

**Published:** 2026-06-25

**Authors:** Patrizia Farci, Davide De Battista, Ronald E. Engle, Eric Chu, Zhaochun Chen, Brendan Jeffrey, Jody Rule, Hanh Nguyen, Sandra Raini, Soichi Takeda, Dean Follman, Harvey J. Alter, William M. Lee

**Affiliations:** 1 Hepatic Pathogenesis Section, Laboratory of Infectious Diseases, National Institute of Allergy and Infectious Diseases, National Institutes of Health, Building 50, Room 6523, 50 South Drive-MSC, Bethesda, MD 20892-8009 USA; 2 Clinical Monitoring Research Program Directorate Frederick National Laboratory for Cancer Research, Frederick, MD USA; 3 Bioinformatics and Computational Biosciences Branch, Office of Cyber Infrastructure and Computational Biology, National Institute of Allergy and Infectious Diseases, National Institutes of Health, Bethesda, 20892 MD USA; 4 Division of Digestive and Liver Diseases, University of Texas Southwestern Medical Center at Dallas, Dallas, TX USA; 5 Biostatistics Research Branch, National Institute of Allergy and Infectious Diseases, National Institutes of Health, Bethesda, MD USA; 6 Department of Transfusion Medicine, Clinical Center, National Institutes of Health, Bethesda, MD USA

**Keywords:** Pathogenesis, HBV, Acute liver failure, Viral factors, Host immunity

## Abstract

**Background:**

Acute hepatitis B virus (HBV) infection encompasses a broad clinical spectrum, from self-limited hepatitis to acute liver injury (ALI), acute liver failure (ALF), subacute liver failure, and ALF on chronic HBV reactivation. This study investigates how host and viral factors interact to shape the clinical spectrum of HBV-associated ALF.

**Methods:**

We analyzed 231 serial serum samples from 49 well-characterized patients including 36 with HBV-associated ALF and 13 with acute hepatitis B. Among ALF patients, 7 had ALI, 7 acute and 11 subacute liver failure, and 11 ALF on chronic HBV. Viral factors, including HBV replication, genotype, sequence diversity and viral evolution by next-generation sequencing (NGS) were assessed alongside host immune responses and cytokine profiles by proteomics.

**Results:**

Demographics were similar across groups. Mortality was highest in subacute liver failure and ALF on chronic HBV (82%) and intermediate in acute liver failure (43%). The three forms of ALF differed by clinical, virologic, and immunologic features. Acute liver failure was characterized by markedly elevated alanine aminotransferase and high IgM anti-HBc titers, whereas ALF on CHB showed higher serum HBV DNA, HBV RNA and HBcrAg levels. HBV genotype A predominated (63%), followed by D (15%), C (9%), and others (2–6%). NGS of the basal core promoter and precore/core regions revealed highly homogeneous viral populations in acute and subacute liver failure throughout follow-up, while ALF on CHB showed marked viral heterogeneity. Genetic diversity was genotype-dependent, unrelated to disease severity, and significantly lower in genotype A than D. Acute liver failure was associated with broad type 1 cytokine response (i.e. IP-10, MIP-1α, MIP-1β, TNF-α, and IFN-γ), restricted type 2 cytokine response (i.e. IL-4 and sCD137), along with proinflammatory (i.e. MCP-1, Il-6, and IL-8) and homeostatic (i.e. IL-7) cytokines, whereas subacute liver failure and ALF on chronic HBV exhibited low and restricted, but distinct cytokine profiles. Elevated GM-CSF and PDGF-BB at admission predicted transplant-free survival.

**Conclusions:**

Distinct clinical, serologic, virologic, and cytokine profiles correlate with disease severity, highlighting differing pathogenic mechanisms across HBV-associated ALF forms. Early biomarkers, including GM-CSF and PDGF-BB, may help predict recovery and guide individualized management to improve survival.

**Supplementary Information:**

The online version contains supplementary material available at 10.1186/s12967-026-08347-z.

## Introduction

Despite the availability of a highly effective vaccine, hepatitis B virus (HBV) infection remains a major global health challenge, affecting over 250 million individuals worldwide, including approximately 2 million in the United States [1]. The clinical outcomes of acute HBV infection range from asymptomatic or self-limited hepatitis to acute liver failure (ALF) and progression to chronic infection. While most of immunocompetent adults clear the virus spontaneously, approximately 1% develop ALF, a rare but often fatal syndrome characterized by massive hepatocyte necrosis in a previously healthy liver, leading to death or the need for liver transplantation in up to 80% of cases [2].

In contrast to acute hepatitis B where liver damage is T-cell mediated [3, 4], the pathogenesis of ALF is still largely unknown due to the dramatic clinical course of this disease, the lack of experimental models, and the difficulties in obtaining samples [2]. Our previous studies provided the first evidence for a distinct immunopathogenic mechanism in HBV-associated ALF, characterized by a dominant humoral immune response [5]. We demonstrated a unique intrahepatic B-cell signature, with extensive production of IgM and IgG antibodies in germline configuration targeting hepatitis B core antigen (HBcAg) with subnanomolar affinity, and complement deposition [6]. HBV strains isolated from ALF livers exhibited highly mutated HBcAg variants, associated with enhanced antigen expression independent of viral replication levels. These findings indicated that humans possess pre-existing germline antibodies against HBcAg and implicate a central role for B cells and viral antigenicity in the pathogenesis of this dramatic clinical syndrome [6]. However, these insights were derived primarily from patients infected with HBV genotype D, enrolled in a single center in Cagliari, Italy. Thus, the extent to which these findings apply to other HBV genotypes, particularly genotype A, which predominates in the United States remains unknown [7]. Furthermore, the clinical presentation of HBV-associated ALF is heterogeneous, ranging from acute to subacute liver failure, and may also result from reactivation or flare of chronic hepatitis B (CHB) [8]. Whether these distinct clinical phenotypes reflect divergent pathogenic mechanisms and immunologic profiles remains unclear.

To address these critical gaps in the pathogenesis of acute liver failure, we studied a unique cohort with serial serum samples obtained from patients across the wide clinical spectrum of HBV-associated acute liver disease including acute and subacute liver failure, and reactivation of CHB leading to ALF. We also included classic acute hepatitis B (without liver failure) as a control group. Our main objective was to investigate the interplay between viral factors–such as HBV genotype, sequence diversity, and viral evolution–and host immune responses, including humoral immunity and cytokine profiles, to better define the mechanisms underlying HBV-associated ALF and its clinical phenotypes, as well as to identify biomarkers predictive of clinical outcomes. Through comparative analyses, this study provides new insights into the pathogenesis of the complex clinical spectrum of HBV-associated ALF and identifies biomarkers that may predict disease outcome.

## Material and methods

### Study population and design

The Acute Liver Failure Study Group (ALFSG) is a multicenter clinical research network sponsored by the National Institute of Diabetes, Digestive and Kidney Diseases that prospectively enrolled patients with ALF or severe acute liver injury (ALI) admitted to 23 different tertiary centers in North America between 1998 and 2019 (see Supplementary Material and Methods for details). ALF was defined by the presence of any degree of hepatic encephalopathy occurring within 26 weeks of symptom onset, coagulopathy defined as an international normalized ratio (INR) >1.5, and no prior history of liver disease. ALI was defined by an INR ≥ 2.0, with no evidence of hepatic encephalopathy. Participants with ALF were enrolled after informed consent was obtained from their legal next of kin or the individual with medical power of attorney, given their altered mentation. For patients with ALI, consent was obtained directly from the patient.

### Determination of ALF etiology and outcome assessment

The principal investigator at each clinical site made the initial determination of causality (etiology of ALF) by reviewing clinical, laboratory, and imaging data available at the time. These diagnoses were subsequently reviewed by a causality committee of experienced hepatologists [9] based on additional information available at a later time point. Patients with acute hepatitis B met the criteria pre-defined by the committee: acute hepatic illness combined with positive tests for IgM anti-HBc and HBsAg in the absence of other apparent etiologies for ALF and no evidence of prior hepatitis B infection. Extensive demographic, laboratory, imaging, tissue sampling when available and clinical data were collected for the first seven days of admission or until orthotopic liver transplantation (OLT), discharge, or death. Outcome analysis was performed at day 21.

Between January 1, 1998, and August 31, 2019, a total of 227 subjects were adjudicated as having HBV-associated ALF (*n* = 168) or ALI (*n* = 59); their ages ranged from 18 to 65 years, and all had known 21-day outcomes. Of these 227 patients, 36 were selected for this intensive study based on the presence of sufficient bio-samples and availability of complete data sets. Among the 36 subjects with HBV-associated ALF, 7 were considered early presentations (Acute) and 11 late presentations (Subacute) based on time from initial symptoms to ALF study enrollment, defined as less than or greater than 10 days [10]; 7 patients presented with ALI. In addition, 11 patients had ALF caused by reactivation of CHB, as determined by prior knowledge or high likelihood of previous HBV infection. Patients were deemed to have HBV reactivation if previously known to be HBV infected, had acute elevation of alanine aminotransferase (ALT), aspartate aminotransferase (AST) and HBV DNA, with negative or very low-titer IgM anti-HBc.

### Classic acute hepatitis B

For comparison, serial serum samples from patients with classic acute hepatitis B without liver failure were obtained from the Hepatitis B Research Network (HBRN) (see Supplementary Material and Methods for details). A total of 60 patients with acute hepatitis B were enrolled and followed for at least 12 months to determine clinical outcomes.

### Serology

Serum hepatitis markers (HBsAg, HBeAg, IgM anti-HBc) were determined using commercially available enzyme immunoassays from Diasorin (Diasorin, Saluggia, Italy) according to the manufacturer’s instructions and quality control procedures. Titer of IgM anti-HBc was obtained by testing 2-fold serial dilutions. Quantitative HBsAg testing was done using a variation of a method described previously [5], as reported in the Supplementary Material and Methods. Antibodies to HBsAg were detected using an enzyme-linked immunosorbent assay (ELISA) Kit (Creative Diagnostics, cat. no. DEIA060) according to the manufacturer’s recommendations.

### HBV core-related antigen

HBV core-related antigen (HBcrAg) quantities were measured using a lumipulse G HBcrAg assay on a lumipulse G1200 analyzer (fujirebio, Tokyo, Japan), as previously described [11].

#### Nucleic acid tests

Serum DNA and RNA were extracted using commercially available kits (DNeasy Blood and Tissue Kit, and QIAamp viral RNA mini kit, Qiagen). Quantification of HBV DNA and HDV RNA in serum was performed by real-time PCR, as previously described [12, 13], and noted in the Supplementary Material and Methods.

#### HBV genotyping

For viral genotype determination, DNA extracted from serum samples was analyzed using a commercially available kit (INNO-LiPA HBV Genotyping Assay, Fujirebio), as detailed in the Supplementary Methods. HBV genotype could not be determined in three patients.

#### Next-generation sequencing of HBV, genome assembly, and sequence analysis

Next-generation sequencing (NGS) was performed on 196 serial serum samples collected from 45 patients. HBV genomes were sequenced from serum-derived DNA, assembled into consensus sequences, genotyped using reference-guided alignment and the Genome Detective HBV Phylogenetic Typing Tool, and analyzed for low-frequency intrahost variants using iVar (See the Supplementary Material and Methods for details).

#### Highly multiplexed codeplex chip-based serum proteomic analysis

Serum cytokine levels were quantified using a multiplex fluorescence ELISA–based platform (IsoPlexis Human Innate Immune Panel) that measured 19 analytes, including Granzyme B, IFN-γ, MIP-1α, TNF-α, GM-CSF, IL-7, IL-8, IL-15, IP-10, MIP-1β, IL-4, IL-10, sCD137, IL-1β, IL-6, MCP-1, EGF, PDGF-BB, and VEGF, as detailed in the Supplementary Material and Methods.

### Statistics

At admission, differences in continuous variables across the five phenotypes were assessed using the Kruskal-Wallis test, followed by Dunn’s multiple comparisons test. Differences in categorical variables among phenotypes were analyzed using the Chi-squared test. The effect of visit, coded as a linear numeric variable, on biomarker values over time within each phenotype group was evaluated using generalized estimating equations (GEE) models, with participants treated as clustering units and an exchangeable correlation structure assumed. To account for the small phenotype group sizes, small-sample adjustments for Wald-type tests were applied using a sandwich estimator of variance from the *saws* package (version 0.9–7.0) [14].

Markers predictive of disease outcome (transplant-free survival versus OLT or death) were identified using Firth’s logistic regression model. Models for each marker included phenotype group as an additive covariate. All the analyses were done on log10 transformed data. To address the presence of zero values in the raw data, a value of 1 was added to all measurements prior to transformation. All *P* values were two-sided. Statistical analyses were conducted using R software, version 4.5.1 (R Foundation for Statistical Computing).

## Results

### Demographic, clinical and virologic features

A total of 49 patients with various acute HBV presentations, including classic acute hepatitis B, ALI, acute and subacute liver failure, and ALF secondary to reactivation (flare) of CHB, were selected for this intensive study (Table 1). These patients represented five distinct phenotypes and clinical outcomes and had serial serum samples available from the day of hospital admission until OLT, death or survival at 21 days. Univariate analysis at hospital admission showed no significant differences in the demographic characteristics among the five groups (Table 1). However, females were predominant among patients with subacute liver failure (64%) compared to those with acute liver failure (43%), whereas males were more prevalent in cases of classic acute hepatitis B (79%) and ALF on CHB (80%). There was no significant difference in the median age among the groups. Notably, we observed a significantly different distribution of mortality rates (defined as OLT or death) among the five groups (*p* < 0.001). In classic acute hepatitis B and ALI none of the patients underwent OLT or died, while subacute liver failure and ALF on CHB showed the highest mortality rates, 82% in both groups. The acute liver failure group showed a mortality rate of 43%, although this rate is based on a limited number of patients included into this intensive study.

**Table 1:** Demographic, clinical, and virological characteristics at admission among five HBV clinical phenotypes

Characteristic	Classic Acute Hepatitis B (n = 13)	ALI^*^ (n = 7)	Acute liver failure			*P *value^**^
			Acute (n = 7)	Subacute (n = 11)	Acute on CHB (n = 11)	
Age, year	34 (31–51)	43 (34–46)	45 (23–53)	33 (31–50)	50 (36–56)	0.285
Male, no. (%)	10 (79)	4 (57)	4 (57)	4 (36)	8 (80)	0.177
*Disease outcome*						
TFS^a^, No. (%)	13 (100)	7 (100)	4 (57)	2 (18)	2 (18)	<0.001
OLT^b^ / Dead, No. (%)	0 (0)	0 (0)	3 (43)	9 (82)	9 (82)	
Alanine aminotransferase, IU/L^c^	2175 (939–3016)	1395 (1233–4895)	2382 (1282–3137)	598 (292–2274)	843 (591–2786)	0.076
Aspartate aminotransferase, IU/L^d^	1186 (746–2208)	1423 (161–2142)	1836 (683–3278)	627 (213–1903)	766 (315–2723)	0.615
Bilirubin, mg/dL^e^	7.6 (4.7–15.7)	20.3 (8.6–24.8)	16.1 (5.5–28.3)	25.6 (19.1–28.8)	14.4 (12.1–19.2)	0.022
INR	1.2 (1.1–1.3)	2.1 (2.1–3.2)	2.6 (2.0–4.5)	3.1 (2.3–3.7)	2.8 (2.4–5.3)	<0.001
*HBV genotype*^f^						
A, no.	9	3	4	9	4	0.115
B, no.	1	0	0	0	2	
C, no.	0	0	1	1	2	
D, no.	3	3	1	0	0	
E, no.	0	0	1	0	0	
G, no.	0	0	0	0	1	
H, no.	0	0	0	0	1	
HBsAg, Log_10_ IU/mL	4.7 (4.0–4.8)	4.6 (0–5.0)	3.7 (1.8–4.6)	3.0 (2.1–4.7)	3.5 (3.3–5.0)	0.503
IgM anti-HBc, Log_10_ titer	5.7 (5.6–5.8)	6.5 (6.3–7.1)	7.0 (5.8–7.3)	5.8 (4.9–6.8)	1.0 (0.3–4.4)	<0.001
*HBeAg*						
Positive, no. (%)	5 (71)	5 (71)	4 (57)	8 (72)	8 (72)	
Negative, no. (%)	2 (29)	2 (29)	3 (43)	3 (28)	3 (28)	0.909
Anti-HBs-positive, no. (%)	0 (0)	3 (43)	1 (14)	1 (9)	1 (9)	0.086
HBV DNA, Log_10_ IU/mL	3.7 (3.2–5.5)	4.2 (3.1–5.5)	3.4 (2.2–6.4)	3.1 (2.9–3.6)	6.8 (3.0–7.0)	0.383
HBcrAg, Log_10_ U/mL	6.5 (6.0–7.0)	5.8 (4.7–6.9)	6.9 (4.3–7.0)	5.5 (5.2–6.0)	7.1 (6.4–7.6)	0.096
HBV RNA, Log_10_ U/mL	0 (0–0)	0 (0–0)	0 (0–3.4)	0 (0–0)	4.2 (2.1–5.5)	<0.001
HDV RNA, Log_10_ U/mL	0 (0–0)	0 (0–0)	0 (0–0)	0 (0–0)	0 (0–0)	-

Quantitative data are shown as median (interquantile range, IQR). ^*^ALI denotes acute liver injury

*P* values were calculated using Kruskal-Wallis for continuous variables and Chi-squared test for categorical variables. ^a^TFS denotes transplant-free survival. ^b^OLT denotes orthotopic liver transplantation. ^c^Normal value, ≤40 IU per L. ^d^Normal value, ≤40 IU per L. ^e^To convert serum bilirubin values to micromoles per liter, multiply by 17.1. ^f^HBV genotype was performed in 46 out of 49 patients, including 6 ALI, 7 with acute and 10 with subacute liver failure, and 10 with acute liver failure on chronic hepatitis B

**Fig. 1:**
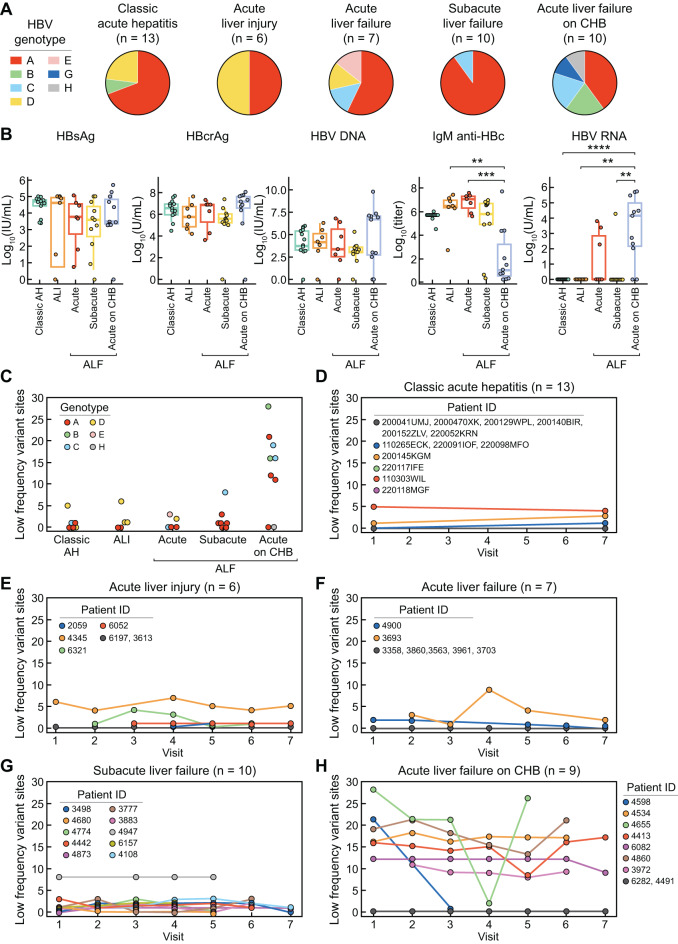
Differences in HBV genotype, serology, virology, and genetic heterogeneity across clinical phenotypes. (**A**) Distribution of HBV genotype among the five clinical phenotypes. (**B**) Serum levels at admission of HBsAg, HBcrAg, HBV DNA, IgM anti-HBc and HBV RNA in patients with classic acute hepatitis B shown in green, acute liver injury (ALI) in orange, acute liver failure (ALF) in red, and subacute liver failure in yellow, and acute liver falure on chronic hepatitis B (CHB) in blue. Box and whisker plots show the median (horizontal line), the interquartile range (25th and 75th percentiles; bottom and top of each box) and the lower and upper whiskers, which cover values within 1.5 × IQR of the first and third quartiles, respectively. (**C**) Number of low-frequency HBV variant sites stratified by clinical phenotype and genotype, assessed both at hospital admission and during longitudinal follow-up. Longitudinal genetic heterogeneity of HBV assessed by next-generation sequencing in serial serum samples from patients with classic acute hepatitis (**D**), acute liver injury (**E**), acute liver failure (**F**), subacute liver failure (**G**), and acute liver failure on CHB (**H**). *p < 0.05, **p < 0.01, ***p < 0.001, ****p < 0.0001, based on Kruskal–Wallis tests followed by Dunn’s test for multiple comparisons

The clinical and virologic characteristics of our cohort at hospital admission are shown in Table 1. Univariate analysis showed that the levels of ALT and AST did not differ significantly among the five groups, although patients with the acute form of ALF exhibited the highest values. Patients with classic acute hepatitis B had the lowest bilirubin and INR values (Kruskal-Wallis test *p* < 0.05 and *p* < 0.001, respectively) consistent with milder liver injury in this group. HBV genotype was determined in 46 out of 49 patients. Most patients were infected with HBV genotype A (29 patients, 63.0%), followed by genotype D (7 patients; 15.2%), genotype C (4 patients; 8.7%), genotype B (3 patients; 6.5%), and one patient each with genotypes E, G, and H (2.2% each) (Table 1 and Fig. 1A).

Notably, virologic profiles distinguished patients with ALF on CHB from the other groups, as they showed the lowest levels of IgM anti-HBc antibodies (*p* < 0.001) but significantly higher serum HBV RNA levels (Table 1 and Fig. 1B; Kruskal-Wallis test *p* < 0.001). No significant differences were observed among the 5 groups for other virologic markers, including HBsAg, HBcrAg and HBV DNA, although their values were consistently higher in patients with ALF on CHB. Serum HDV RNA was negative in all patients (Table 1).

### Genetic characterization of HBV strains by next generation sequencing in patients with different clinical phenotypes and HBV genotype

To investigate the genetic characteristics of HBV strains associated with various clinical forms of acute HBV infection, we performed NGS on serial serum samples from 45 patients. After PCR amplification of an 877-nucleotide region encompassing the basal core promoter (BCP) and precore/core (preC/C) regions (nucleotides 1624–2457), 196 HBV DNA samples were sequenced. These included samples from 13 patients with acute hepatitis B, 6 with ALI, 17 with ALF (7 acute and 10 subacute) and 9 with ALF on CHB. NGS yielded over 10^6^ reads per sample. Sequence analysis documented that patients with acute and subacute liver failure harbored a highly homogeneous viral population (>99%), while ALF on CHB had the greatest viral heterogeneity (Fig. 1C). Notably, few or no sequence changes were observed in serial serum samples from patients with classic acute hepatitis (Fig. 1D), ALI (Fig. 1E), acute liver failure (Fig. 1F) and subacute liver failure (Fig. 1G) collected at different time points after hospital admission, suggesting genetic stability during the acute disease phase. In contrast, more variability was seen in ALF on CHB (Fig. 1H).

**Fig. 2:**
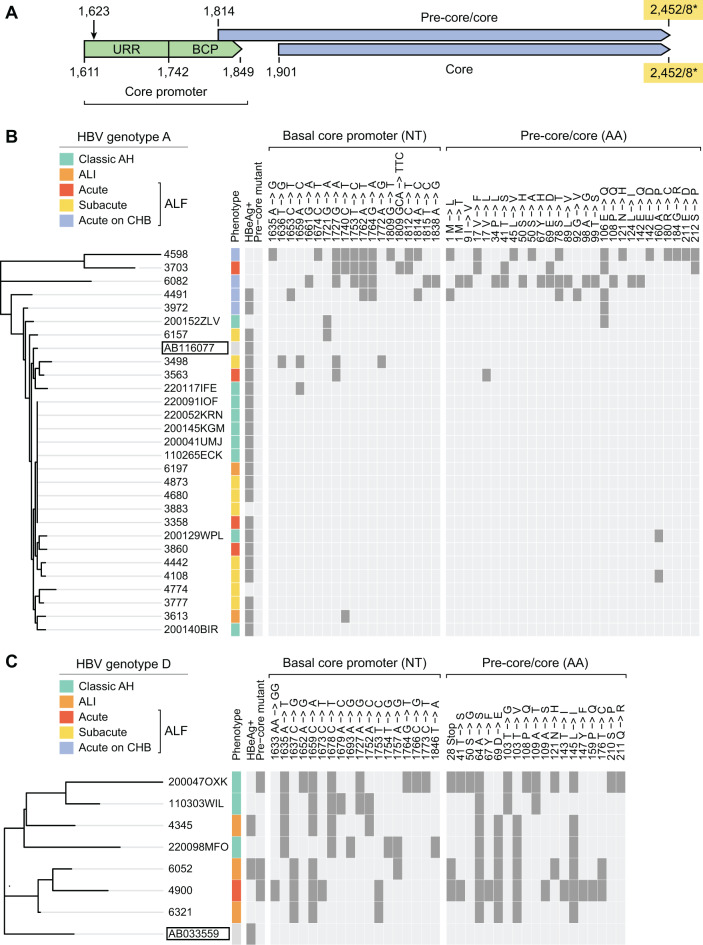
Gene structure and genotypic variations in the basal core promoter and pre-Core/Core regions. (**A**) Schematic representation of the basal core promoter and pre-core/core protein regions, with nucleotide coordinates shown for genotype A (AB1160777) and genotype D (AB033559). The pre-core/core region terminates at nucleotide 2458 for genotype A and at nucleotide 2452 for genotype D, as indicated by the asterisk. The arrow indicates the start of the PCR product for sequencing analysis (nucleotides 1624–2457). (**B**, **C**) Genotype-specific variations for genotype A (**B**) and genotype D (**C**) relative to their respective reference genomes (indicated by the boxes). Nucleotide substitutions in the basal core promoter are presented as nucleotide changes, whereas variations within the pre-core/core region are shown as amino acid substitutions

Accurate sequence analysis of the BCP and preC/C regions (Fig. 2A) requires genotype-specific reference genomes to avoid misclassification of nucleotide substitutions. Because genotype A includes highly divergent subgenotypes and all genotype A samples in this U.S cohort belonged to subgenotype A2, we selected AB116077, a U.S derived genotype A2 sequence [15] as the reference after comparison with other available genotype A sequences (e.g., X70185). For genotype D, the standard reference sequence AB033559 was used.

Analysis of specific mutations showed striking differences among HBV genotypes. In HBV genotype A-infected patients, NGS showed that the precore stop codon mutation G1896A, which abolishes HBeAg production, was absent at admission and during follow-up (Fig. 2B). Dual BCP mutations (A1762T/G1764A) were detected exclusively in three ALF-on-CHB cases and one acute liver failure case. These patients were HBeAg-negative and harbored additional mutations in the BCP and preC/C regions (Fig. 2B). Loss of HBeAg in these cases was likely due to disruption of precore translation, caused by mutations affecting the initiation codon (ATG), including a TCC substitution at position 1812 and A1814C and T1815C changes. In contrast, most genotype A–infected patients, regardless of clinical phenotype or HBeAg status, lacked the dual BCP mutations and showed few or no additional changes (Fig. 2B), suggesting these mutations are not essential for the development of HBV-associated ALF.

In genotype D–infected patients, numerous BCP and preC/C mutations were observed, but these were not associated with clinical phenotype, HBeAg status, or the presence of G1896A or dual BCP mutations (Fig. 2C), indicating that mutation patterns in these regions are largely genotype-dependent rather than ALF-specific.

Other HBV genotypes were uncommon in our cohort, consistent with the predominance of genotype A in the United States. Only three ALF cases involved genotype C or E, whereas in ALF on CHB, six of ten patients carried genotypes other than A or D (Table 1). No genotype-specific mutation patterns associated with ALF were identified. Among these less common genotypes, diverse combinations of BCP and preC/core mutations were observed (Supplementary Table 1), irrespective of genotype or disease phenotype. Overall, these findings indicate that mutations in the BCP and preC/C regions are not specifically associated with HBV-associated ALF.

### Distinct cytokine profiles correlated with disease severity and clinical outcome

The absence of genotype-specific mutations associated with the various forms of acute HBV infection, particularly within genotype A, which predominated in both classic acute hepatitis and in acute and subacute liver failure, prompted us to investigate the role of host factors contributing to the pathogenesis of disease severity. Because viable cellular samples were unavailable from this unique cohort of patients, we assessed virus-elicited immune responses by analyzing the long-term profile of 19 cytokines in daily serial serum samples using an automated proteomic approach. IL-15 was excluded, as it was below the detection threshold in most samples (188 of 208; 90.4%). Cytokines were grouped as type 1 (IP-10, MIP-1α, MIP-1β, granzyme B, TNF-α, IFN-γ, and IL-1β), type 2 (IL-4, sCD137), other cytokines, including proinflammatory (IL-6, IL-8, and MCP-1), homeostatic (IL-7), immune-regulatory (IL-10) and growth factors (GM-CSF, PDGF-BB, EGF, VEGF). Serum levels at hospital admission in our patient cohort are presented in Table 2.

**Table 2:** Median serum cytokine levels at admission across five HBV clinical phenotypes

Characteristic	Classic Acute Hepatitis B (n = 13)	ALI^*^ (n = 7)	Acute Liver Failure			*P *value^**^
			Acute (n = 7)	Subacute (n = 11)	Acute on CHB (n = 8)	
*Type 1 immunity*						
Granzyme B	0 (0–0)	28.5 (3.5–220.2)	84.3 (18.9–1043.4)	0 (0–2.2)	0 (0–13.8)	0.047
IFN-γ	11.3 (0–19.2)	0.5 (0–13)	17.2 (8.1–65.7)	0 (0–0)	0 (0–1.6)	0.112
IL-1β	0 (0–0)	0 (0–0.3)	5 (0–7.7)	0 (0–0.1)	0 (0–0.4)	0.324
IP-10	11,883.6 (2212–17009.6)	7190.4 (1339.9–9629.7)	4289.2 (3694–6978.8)	764.2 (360.4–2731.7)	268.4 (73.8–3065.9)	0.006
MIP-1α	365.4 (223.1–508.9)	409.4 (128.9–581.4)	1118.3 (615.9–2513.8)	361.2 (186.5–558.4)	31.6 (5.8–88.3)	0.002
MIP-1β	1578.7 (1079–1629.3)	830.1 (371.4–1181.1)	829.9 (392–1006.1)	1230.1 (550.3–1570)	719.9 (362–1466.6)	0.077
TNF-α	0 (0–0)	19.7 (0.8–59.6)	34.7 (13.8–121.6)	0 (0–0)	0.2 (0–8)	0.003
*Type 2 immunity*						
IL-4	12.3 (0.3–53.7)	1293.5 (12.9–1387.7)	1304.9 (750.2–2181.2)	38.3 (0–139.3)	191.6 (115.8–640)	0.027
sCD137	0 (0–53.8)	361.8 (0–536.9)	704.2 (373.2–906.3)	0 (0–17.5)	247.8 (146.4–843.9)	0.002
*Other cytokines*						
IL-6	0 (0–31.7)	99.4 (21.9–201.2)	118 (83.1–186.6)	57 (27.9–97)	62.3 (40–260.2)	0.006
IL-7	110.4 (43.9–177.2)	155.9 (107.8–476.4)	587.2 (353.3–720.3)	35.2 (7.4–139.3)	35.3 (0–597.7)	0.029
IL-8	0 (0–0)	0 (0–22.7)	0 (0–26.6)	0 (0–0)	395.8 (0–823.4)	0.011
IL-10	22.8 (11.9–28.4)	10.7 (4.9–14.2)	32.1 (19–155)	9.5 (3.8–25)	13.7 (2.4–26.2)	0.129
MCP-1	654.1 (70.6–783.9)	715.7 (501.7–1190.3)	1845.1 (838.2–4001.2)	1233.8 (632.7–1837)	653.8 (335.1–1648.5)	0.163
*Growth factors*						
EGF	373.2 (118–462.8)	336.9 (152.2–666.6)	88.8 (34.6–206.4)	72.3 (49.8–335.9)	67.5 (26.9–180.5)	0.146
GM-CSF	0 (0–0)	0 (0–0)	0.6 (0–7.3)	0 (0–0)	0 (0–0.5)	0.074
PDGF-BB	61,149.5 (54676–76041)	34,644.9 (17268–71121.9)	25,818.2 (13676–45288.9)	56,929.7 (27681.3–65643.4)	4498 (2795.7–13231.6)	0.007
VEGF	116.8 (13.6–493)	34.6 (8.1–461.3)	56.1 (5.3–218.6)	33.3 (6.3–298)	8.5 (0–54.3)	0.199

Quantitative data are presented as median (interquantile range, IQR). All concentrations are expressed in pg per mL. ^*^ALI denotes acute liver injury ^**^P values were calculated using the Kruskal-Wallis test

**Fig. 3:**
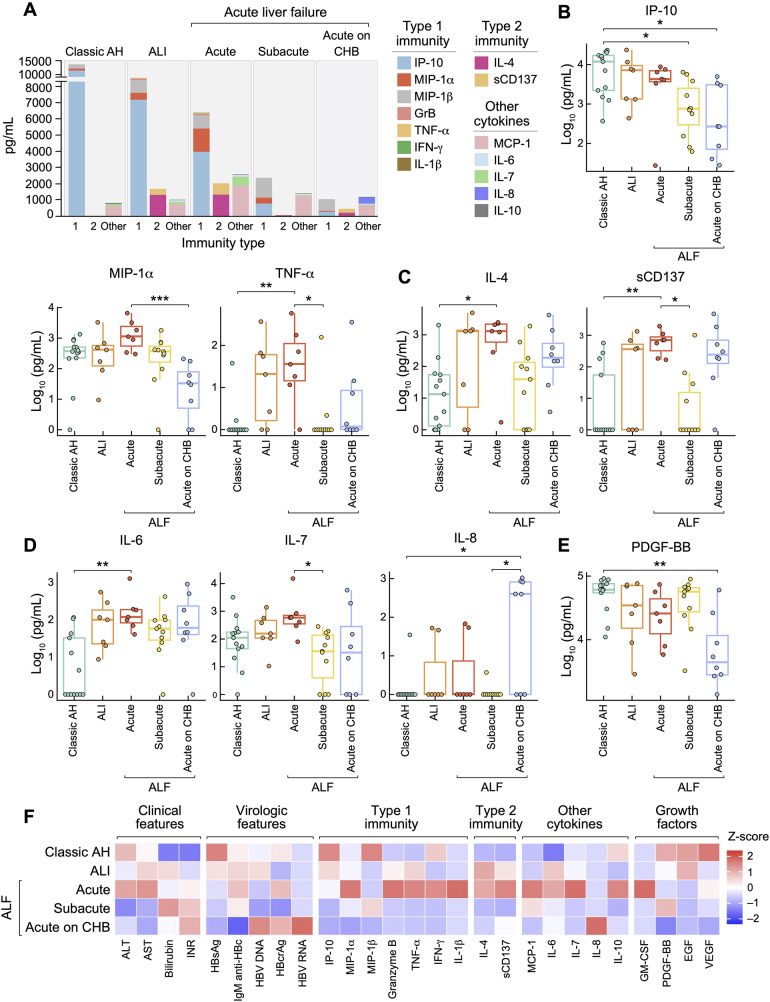
Distinct cytokine profiles associated with disease severity and clinical outcome across the five clinical phenotypes at hospital admission. (**A**) Stacked bars comparing the median serum levels of type 1 and type 2 cytokines measured in each group using a proteomic approach. (**B**, **C**) Serum levels of type 1 (B), type 2 (C) immunity-related cytokines, other cytokines (**D**) and growth factors (**E**) showing statistically significant differences among the five clinical phenotypes: classic acute hepatitis (AH) (green), ALI (orange), acute liver failure (red), subacute liver failure (yellow), and ALF on CHB (blue). Box- and-whisker plots show the median (horizontal line), the interquartile range (25th and 75th percentiles; bottom and top of each box) and the lower and upper whiskers, which cover values within 1.5 × IQR of the first and third quartiles, respectively. (**F**) Heatmaps comparing median values of clinical and virological features, serum cytokines representing type 1 and type 2 immunity, and growth factors across the five clinical phenotypes. ALF denotes acute liver failure; CHB denotes chronic hepatitis B. Data were normalized using Z-scores. For each biomarker, the Z-score for each phenotype group was calculated as the group median minus the overall mean for that biomarker, divided by the overall standard deviation. *p < 0.05, **p < 0.01, ***p < 0.001, based on Kruskal–Wallis tests followed by Dunn’s test for multiple comparisons

Comparison of the 5 clinical groups revealed distinct cytokine profiles associated with disease severity and clinical outcomes. At admission, patients with classic acute hepatitis B showed a dominant type 1 immune response, with early induction of IP-10, MIP-1α and MIP-1β (Fig. 3A and B), which are key mediators of type 1 inflammation. IP-10, an interferon-inducible CXC motif chemokine, plays a critical role in T cell recruitment and in the orchestration of Th1-type immune responses. In contrast, the expression of type 2 cytokines was minimal. The broadest cytokine response was observed in patients with acute liver failure, characterized by marked increases in type 1 cytokines, particularly MIP-1α and TNF-α (Fig. 3A, B), as well as a type 2 response, including remarkably elevated levels of IL-4 and sCD137 (Fig. 3C). These patients also showed high levels of the proinflammatory cytokine MCP-1 and the homeostatic cytokine IL-7 (Fig. 3D), compared to the other groups (Fig. 3A, D, Supplementary Figure 1). Patients with ALI exhibited an intermediate cytokine profile between classic acute hepatitis B and acute liver failure, with predominance of type 1 immune response (Fig. 3A-D, Supplementary Figure 1). In contrast, patients with subacute liver failure and ALF on CHB (Table 1), showed a markedly restricted cytokine response (Fig. 3A-D, Supplementary Figure 1A and B), although proinflammatory cytokines remained detectable (Fig. 3E, Supplementary Figure 1C). Notably, ALF on CHB was uniquely characterized by elevated IL-8 (CXCL8) levels (Fig. 3D), a potent proinflammatory chemokine associated with neutrophil recruitment and tissue infiltration. Regarding growth factors, PDGF was the only factor showing a significant difference, with significantly higher levels observed in classic acute hepatitis B compared with acute-on-CHB cases, whereas similar levels were found among the remaining groups (Fig. 3E).

The cytokine profile observed in acute liver failure correlated with the highest ALT, AST, and IgM anti-HBc levels, consistent with a strong immune-mediated liver injury (Fig. 3F). Conversely, ALF on CHB showed the highest HBV DNA, HBcrAg, and HBV RNA levels, together with the lowest IgM anti-HBc titers indicating active viral replication (Figs. 1B and 3F). Notably, HBV RNA levels were significantly elevated and predominantly detected in ALF on CHB compared to the other clinical groups.

### Comparative analysis among the three main forms of HBV-Associated ALF

Our study highlights the broad clinical spectrum of ALF, revealing distinct patterns that correlate with disease severity. Differences in mortality among ALF subtypes, despite infection with the same HBV genotype and irrespective of BCP or preC/C mutations, suggest the involvement of distinct pathogenic mechanisms. To explore these mechanisms, we compared the three major HBV-ALF subtypes to identify factors associated with pathogenesis and clinical outcomes.

**Fig. 4:**
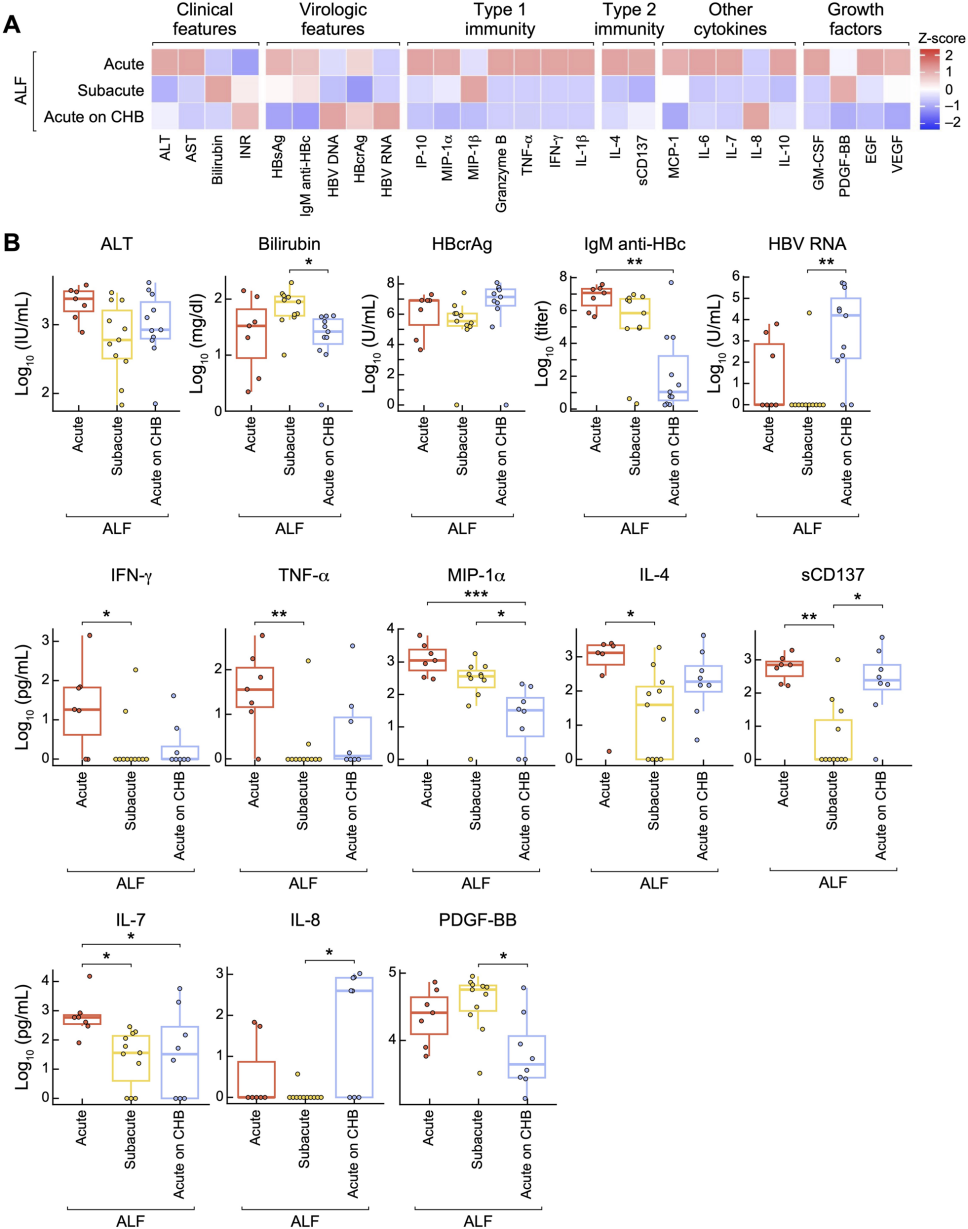
Distinct cytokine profiles correlated with disease severity and clinical outcome among the three forms of acute liver failure at admission. (**A**) Heatmaps comparing the median values of biochemical and virological features, and serum levels of type 1 and type 2 cytokines, other cytokines, and growth factors across acute liver failure, subacute liver failure, and ALF on CHB. Data were normalized using Z-scores. For each biomarker, the Z-score for each phenotype group was calculated as the group median minus the overall mean for that biomarker, divided by the overall standard deviation. (**B**) Box and whisker plots show the median (horizontal line), the interquartile range (25th and 75th percentiles; bottom and top of each box), and the lower and upper whiskers, which extend to the smallest and largest values within 1.5 × IQR of the first and third quartiles, respectively, for clinical, virological, and cytokine parameters that significantly differ among acute liver failure (red), subacute liver failure (yellow), and acute liver failure on CHB (blue). *p < 0.05, **p < 0.01, ***p < 0.001, based on Kruskal-Wallis tests followed by Dunn’s test for multiple comparisons

Acute and subacute liver failure differed in clinical and immunologic features. Acute liver failure was associated with significantly higher peak ALT levels than subacute liver failure (median 2,382 vs. 598 IU/L; Table 1; Fig. 4A and B). Cytokine profiling documented that acute liver failure exhibited markedly elevated type 1 (IFN-γ, TNF-α) and type 2 cytokines (IL-4, sCD137) along with IL-7, whereas subacute liver failure showed a more restricted cytokine response (Fig. 4A and B).

**Fig. 5:**
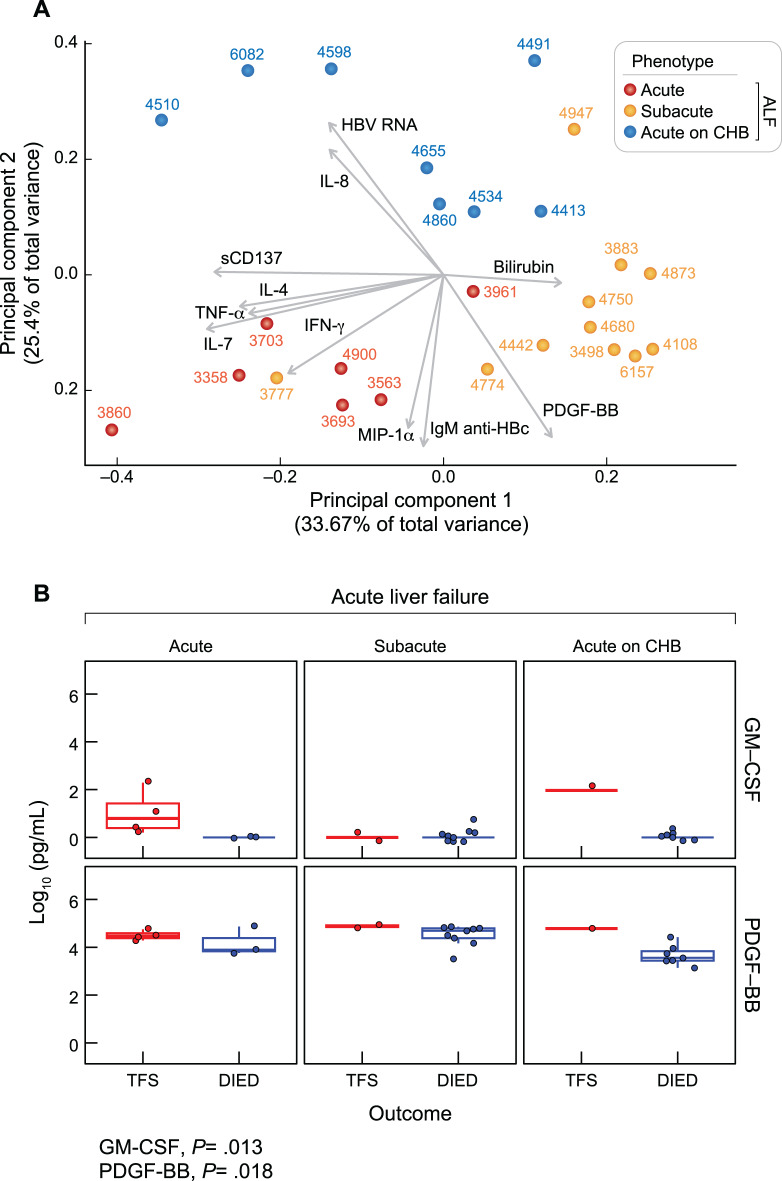
Principal component analysis (PCA) reveals distinct serologic, virologic, and cytokine signatures among liver failure phenotypes and predictive factors associated with transplant free survival (TFS). (**A**) PCA biplot generated from serologic, virologic, and cytokine variables that differed significantly across phenotype groups including acute liver failure, subacute liver failure, and acute liver failure on CHB, by Kruskal–Wallis testing (*p* < 0.05). Each point represents an individual patient and is colored according to the clinical phenotype. Principal component 1 explains 33.67% of the total variance and principal component 2 explains 25.36%. The biplot demonstrates a clear separation among phenotype groups along the first two principal components. Vectors indicate the relative contribution of each variable, with direction and length reflecting the extent of their association with the principal components. (**B**) Predictive factors associated with transplant free survival (TFS) are shown stratified by the three forms of acute liver failure. Box-and-whisker plots show the median (horizontal line), the interquartile range (25th–75th percentiles; box boundaries), and the lower and upper whiskers, which cover values within 1.5 × IQR of the first and third quartiles, respectively. *P* values were calculated by Firth’s logistic regression model

Compared with ALF on CHB, acute liver failure showed significantly higher IgM anti-HBc and IL-4 levels, indicating a stronger humoral immune response. In contrast, subacute liver failure differed from ALF on CHB by higher bilirubin and markedly lower HBV RNA levels, consistent with reduced viral replication. It was further characterized by elevated MIP-1α and PDGF-BB, but lower IL-8 and sCD137 levels (Fig. 4A and B, Supplementary Figure 2). Collectively, our findings demonstrate that the three forms of ALF exhibit distinct clinical, virologic, and immunologic profiles, as evidenced by their clear separation in the principal component analysis (PCA) (Fig. 5A).

### Longitudinal analysis of clinical, virological, and cytokine expression

Longitudinal changes were assessed in 227 serial serum samples, with a median of 2 time points (range, two to three) for classic acute hepatitis B and 5 time points (range, 3 to 7) for the other 4 groups. Temporal trends in clinical, virological, and cytokine parameters were analyzed using GEE models.

In classic acute hepatitis B, clinical parameters (ALT, AST, bilirubin) and virological markers (HBsAg, IgM anti-HBc, HBV DNA, HBcrAg) gradually normalized over time (Supplementary Table 2, Supplementary Figure 3), accompanied by a decline in type 1 cytokines (IP-10, MIP-1β, IFN-γ) and the homeostatic IL-7 cytokine. In acute liver failure, HBV DNA levels decreased over time, along with MIP-1α, granzyme B, IL-4, and sCD137 through day 6. In contrast, subacute liver failure showed a decline in IP-10 but a notable increase in TNF-α levels. Other variables did not show significant changes over time within any group (Supplementary Table 2, Supplementary Figure 3).

### GM-CSF and PDGF-BB predict transplant-free survival in acute liver failure

To identify predictors of transplant free survival across the three forms of ALF–acute liver failure, subacute liver failure and ALF on CHB – and adjust for the small group sizes, we applied Firth’s logistic regression model. This analysis identified two markers significantly associated with transplant free survival (Fig. 5B). Patients who survived without requiring OLT had significantly higher circulating levels of granulocyte-macrophage colony-stimulating factor (GM-CSF) (*p* = 0.013) and platelet-derived growth factor subunit BB (PDGF-BB) (*p* = 0.018). Both of these analytes are classified as growth factors and may play a protective or regenerative role in the context of acute liver injury.

## Discussion

The diverse clinical presentations of acute HBV infection, ranging from primary acute self-limited hepatitis B to ALI, acute and subacute presentations of ALF, and ALF secondary to acute exacerbation (flare) of CHB are thought to result from complex host–virus interactions. However, the mechanisms that drive these divergent outcomes remain poorly understood. By integrating viral and host analysis, our study provides new insights into the clinical, virological, and immunological determinants that shape the heterogeneous manifestations of HBV-associated ALF. Importantly, our cohort was free from major confounders, including coinfection with HDV or HCV, intravenous drug use, and alcohol abuse. The predominance of genotype A (63%) also allowed exploration of genotype-specific effects, addressing a gap in prior studies largely focused on other HBV genotypes [5, 6].

Comparative analysis of biochemical parameters across clinical phenotypes showed a significant difference only in a coagulopathy marker (INR), which was most aberrant in patients with subacute liver failure. ALT levels did not differ significantly between groups, though the lowest median ALT levels were seen in subacute liver failure. In classic acute hepatitis B and ALI, none of the patients underwent OLT or died. In contrast, those with acute liver failure had a mortality rate of 43% and those with subacute liver failure and ALF on CHB had a higher mortality rate (82% in both groups). These percentages should be interpreted with caution, as they are derived from a limited number of selected patients and reflects the nature of an intensive pathogenesis-focused study rather than a large population-based study.

Distinct virological features were also identified among the groups. The most notable were the remarkably higher IgM anti-HBc titers in acute liver failure and elevated serum HBV RNA levels in ALF on CHB. Although other serologic and virologic features did not differ significantly across groups, serum HBV DNA levels tended to be higher in ALF on CHB, whereas HBsAg titers were higher in classic acute hepatitis B and ALI. These observations suggest that disease severity was not directly associated with the magnitude of HBV DNA replication, consistent with previous reports [5, 6, 8, 16, 17].

NGS analysis of HBV strains demonstrated that patients with acute and subacute liver failure, ALI, and classic acute hepatitis B harbored highly homogeneous viral populations at admission, which remained stable throughout longitudinal follow-up. In contrast, patients with ALF secondary to reactivation of CHB exhibited the greatest degree of viral heterogeneity, consistent with the accumulation of mutations during long-standing infection. Previous studies have reported strong associations between HBV-associated ALF and specific viral mutations, including the precore stop codon mutation (G1896A) and the dual BCP mutations A1762T/G1764A [17–20]. Our earlier work similarly demonstrated highly mutated viral strains in patients with acute HBV ALF harboring HBV genotype D [6]. However, these and other studies have emphasized that the prevalence and functional impact of these mutations are strongly genotype dependent. For example, the precore stop codon mutation (G1896A) is rarely detected in genotype A, as it destabilizes the hairpin RNA structure of the encapsidation signal, leading to deleterious effects on viral replication [21–23]. Indeed, several studies have consistently reported the absence of G1896A in HBV genotype A, regardless of disease outcome and severity [24, 25]. In agreement with these findings, our current study identified several precore mutations among patients infected with genotype D, whereas the canonical G1896A mutation was not detected in those infected with genotype A. Instead, we discovered novel mutations affecting the precore initiation codon (ATG), which was mutated to either CTG or ACT (Fig. 2B), thereby abolishing HBeAg production through a mechanism different from the well-known G1896A precore stop codon mutation. These mutations were present in only one acute liver failure case and in two patients with ALF on CHB. Notably, in these few cases that exhibited extensive mutations within the BCP and preC/C regions, the dual A1762T and G1764A mutations were consistently present. In contrast, these dual mutations were absent in most cases with few or no genetic changes, suggesting a relationship between the presence of BCP/preC/C dual mutations and the overall extent of viral genetic diversity. Our larger and genotypically diverse cohort provides strong evidence that mutations in the BCP and preC/C regions are not uniquely linked to the pathogenesis of HBV-associated ALF, but instead reflect broader genotype-dependent patterns of viral evolution.

The fact that most patients were infected with the same HBV genotype along with the absence of specific BCP and preC/C mutations, underscore the critical role of host factors and point to distinct pathogenic mechanisms driving disease severity. HBV is generally considered a noncytopathic virus [26]. In classic acute hepatitis B, liver injury and viral clearance are mediated by robust, virus-specific antiviral T-cell responses [27], which eliminate infected hepatocytes through both cytolytic and noncytolytic, cytokine-mediated, mechanisms [26–28] involving interferon gamma (IFN-γ) and tumor necrosis factor alpha (TNF-α) [29]. To investigate the basis for the striking differences in disease severity following primary acute HBV infection, we compared the cytokine profiles in classic acute hepatitis B, ALI, acute and subacute liver failure, and in ALF secondary to reactivation of CHB. Because viable cellular samples were unavailable to study virus-specific T-cell responses, we assessed virus-elicited immune responses by measuring 19 cytokines in serial serum samples collected at hospital admission and longitudinally until patients either recovered without OLT, underwent transplantation or died. We found that type 1 immunity, characterized by IFN-γ-inducible cytokines, with IP-10 being dominant, was highest in classic acute hepatitis B. Notably, type 1 responses declined progressively from classic acute hepatitis to ALI and ALF, whereas type 2 immune responses increased with worsening disease severity (Fig. 3A). Interestingly, acute ALF was characterized by a simultaneous and abundant elevation of both type 1 and type 2 cytokines, along with the highest expression of the proinflammatory cytokine MCP-1. In contrast, subacute liver failure and ALF on CHB, exhibited uniformly low but distinct cytokine signatures, consistent with a state of immune dysfunction or exhaustion, with minimal expression of type 2 cytokines rather than a simple shift from type 1 to type 2 immunity.

Our study also provides evidence that HBV-associated ALF is not a single, uniform disease entity but rather encompasses a heterogeneous spectrum of clinical presentations. This heterogeneity implies that different pathogenic mechanisms may be operating across patients. Recognizing this spectrum is clinically important because it highlights that management strategies and prognostic indicators may need to be tailored to the underlying mechanism rather than applied uniformly across all HBV-related ALF cases. First, our data show that acute and subacute liver failure exhibit distinct outcome-specific features that are already apparent at hospital admission. Acute liver failure is characterized by significantly higher ALT levels and a broad induction of both type 1 and type 2 cytokine responses, including significantly elevated levels of IFN-γ, TNF-α, IL-4, sCD137, as well as the homeostatic IL-7 cytokine, which was recently associated with liver fibrogenesis in a NAFLD mouse model [30]. Given the critical role of IL-4 in promoting B-cell activation and humoral immunity [31], we found that high IL-4 levels correlated with the highest titers of IgM anti-HBc. This observation is consistent with our previous work, in which comprehensive genetic and molecular analysis of serum and liver samples from four patients with HBV-associated ALF demonstrated a major role for the humoral immunity in the pathogenesis of this disease [5, 6]. Gene expression profiling documented an overwhelming B-cell gene signature in the liver of patients with acute liver failure, with extensive expression of immunoglobulin genes and genes involved in B-cell development, maturation and survival. This was accompanied by massive intrahepatic accumulation of plasma cells secreting IgG and IgM in germline configuration exclusively targeting HBcAg with subnanomolar affinity, and complement deposition [5, 6]. Although liver biopsies were not available from the present cohort of patients, the combination of the highest IgM anti-HBc titers and robust IL-4 response strongly supports a major role for the humoral immunity in the pathogenesis of hepatitis B-related rapidly evolving ALF. In contrast, a distinct picture emerged for subacute liver failure that, unlike the acute form of ALF, was characterized by a restricted and overall low expression of both type 1 and type 2 immune responses. IL-4 levels were significantly lower in subacute liver failure, as were IL-7 and sCD137, whereas IFN-γ and TNF-α levels were undetectable in nearly all patients. The coexistence of well-represented type 1 and type 2 responses was therefore a hallmark of acute liver failure.

One of the most important advances in recent years was the discovery that group 2 innate lymphoid cells (ILC2s), which are resident cells, constitute a critical innate source of type 2 cytokines and do so even in the absence of adaptive immunity [30]. Given the dramatic clinical course of acute liver failure, we hypothesize that patients who survive without OLT may reflect the ability of certain cytokines, such as MCP-1, IL-6 and IL-8 to simultaneously drive inflammation and support tissue repair [31]. In line with this hypothesis, prior work demonstrated that ALF is associated with hepatic stellate cell activation, a progenitor cell response, and increased liver stiffness, suggesting that fibrosis is a response to ALF in an attempt to repair acute tissue injury [32, 33]. Further studies using liver tissue from ALF patients are warranted to investigate the delicate balance between inflammation and tissue repair, and how these processes differ between individuals who survive without transplantation and those who die or require OLT.

Another interesting observation from our study was that, although the mortality rates for subacute liver failure and ALF on CHB were similarly high (>80%), these two forms showed distinct clinical, virologic and immunologic characteristics (Fig. 5A), suggesting that they arise from different underlying pathogenic mechanisms. ALF on CHB was characterized by significantly higher levels of HBcrAg, HBV RNA, sCD137, IL-8, and low IgM anti-HBc levels. The elevated expression of IL-8, a potent proinflammatory chemokine that recruits and activates neutrophils and monocytes, points to a distinct mechanism of liver injury in this group, consistent with the hypothesis that liver damage is the result of massive infiltration and activation of nonspecific inflammatory cells and continuous release of proinflammatory mediators. Prior studies of patients with decompensated cirrhosis have shown that those who develop acute-on-chronic liver failure (ACLF) display marked dysregulation of circulating immune cells, including neutrophilia and depletion of several lymphocyte subsets, such as memory lymphocytes [34, 35]. These lymphocyte abnormalities, together with impaired neutrophil superoxide anion production, are thought to contribute to the immunosuppressed state characteristic of ACLF [34, 35].

Our longitudinal study also provides new insights into biomarkers that may predict transplant-free survival across acute liver failure, subacute liver failure and ALF on CHB. We found that elevated circulating levels of GM-CSF and PDGF-BB at admission were independently predictive of transplant-free survival. The strength and consistency of these associations, confirmed through additional modeling using Firth’s logistic regression to address separation and small-sample bias, highlight both GM-CSF and PDGF-BB as promising prognostic biomarkers. GM-CSF is recognized as an immune-modulating cytokine that may contribute to improved outcomes through its immune-modulating and immune-restoring activities, as well as by supporting liver regeneration, facilitating tissue repair, and enhancing infection control [36–38], whereas PDGF-BB may influence transplant-free survival through a complementary role in liver repair. As one of the most potent mitogens for hepatic stellate cells, PDGF-BB supports the structural and microenvironmental changes needed for liver repair [39, 40], which may reflect or promote a more effective regenerative response in patients who ultimately recover without liver transplantation. Together, these findings suggest that integrating both GM-CSF and PDGF-BB into risk-prediction models may enhance early stratification of patients and improve identification of those with greater potential for spontaneous recovery. In a recent study, increased levels of angiogenic factors from admission, including PDGF-BB, were reported to be associated with transplant-free survival in patients with acute liver failure [41]; however, the study did not distinguish between acute and subacute forms of HBV-associated ALF, nor did it differentiate acute-on-chronic HBV-related ALF.

In summary, our study highlights the broad clinical spectrum and variable outcomes of HBV-associated ALF and identify distinct clinical, serologic, virologic, and cytokine profiles that not only correlate with disease severity and outcome but also suggest differing pathogenic mechanisms across the three major forms. Importantly, the early identification of prognostic biomarkers, such as GM-CSF and PDGF-BB, at hospital admission may allow stratification of patients according to their likelihood of spontaneous recovery versus progression to liver failure requiring OLT. This approach has the potential to support more precise, individualized management, by optimizing the timing of interventions and potentially improving survival in this high-risk population. Further studies in large cohorts will be necessary to confirm our findings and to define their broader clinical and translational relevance in HBV-associated acute liver failure. Although difficult to obtain, liver biopsies from patients with acute liver failure would be invaluable for investigating host-virus interactions at the site of disease.

## Electronic supplementary material

Below is the link to the electronic supplementary material.

Supplementary Material 1

## Data Availability

Next-generation sequencing data of HBV strains have been deposited in the National Center for Biotechnology Information (NCBI) Sequence Read Archive (SRA)(accession no. PRJNA1392027) and are publicly available as of the date of publication.

## Abbreviations

ALF: Acute liver failure
ALFSG: Acute Liver Failure Study Group
ALI: Acute liver injury
Anti-HBc: Hepatitis B core antibody
Anti-HBe: Hepatitis B e antigen antibody
Anti-HBs: Hepatitis B surface antibody
ALT: Alanine aminotransferase
AST: Aspartate aminotransferase
BCP: Basal core promoter
CHB: Chronic hepatitis B
HBRN: Hepatitis B Research Network
HBV: Hepatitis B virus
EGF: Epidermal growth factor
ELISA: Enzyme-linked immunosorbent assay
GEE: Generalized estimating equations
GM-CSF: Granulocyte-macrophage colony-stimulating factor
HBcAg: Hepatitis B core antigen
HBcrAg: Hepatitis B core related antigen
HDV: Hepatitis delta virus
HBeAg: Hepatitis B e antigen
HBsAg: Hepatitis B surface antigen
IFN: Interferon
IL: Interleukin
INR: International normalized ratio
MCP-1: Monocyte chemoattractant protein-1
MIP: Macrophage inflammatory protein
NGS: Next generation sequencing
OLT: Orthotopic liver transplantation
PCR: Polymerase chain reaction
PDGF-BB: Platelet-derived growth factor-BB
PreC/C: Pre-core/core region
sCD137: Soluble CD137
TNF-α: Tumor necrosis factor-α
VEGF: Vascular endothelial growth factor
