# Supplementary material for: Role of viral and host factors in determining the outcome of HBV-associated acute liver failure
Source: J Transl Med. 2026 Jun 25;24:836. doi: 10.1186/s12967-026-08347-z (PMC13326087; doi:10.1186/s12967-026-08347-z)
Supplement: Supplementary file 1 — Supplementary Material 1 [file 12967_2026_8347_MOESM1_ESM.docx]

**Supplementary Information**

**Role of viral and host factors in determining the outcome of**

**HBV-associated acute liver failure**

**Patrizia Farci^1*^, Davide De Battista^1^**^†^**, Ronal E. Engle^1^**^†^**, Eric Chu^2^, Zhaochun Chen^1^,**

**Brendan Jeffrey,^3^ Jody Rule^4^, Hanh Nguyen^1^, Sandra Raini^1^, Soichi Takeda^1^,**

**Dean Follmann^2^, Harvey J. Alter^5^, William M. Lee^4^**

*****Corresponding author. Email: [pfarci@niaid.nih.gov](mailto:pfarci@niaid.nih.gov)

^†^**Contributed equally**

**METHODS**

# **Patients**

**The Acute Liver Failure Study Group (ALFSG)**

The ALFSG was established in 1998 as a prospective registry collecting detailed de-identified information, including demographic, clinical, laboratory, imaging, and outcome data, along with serial biospecimens, from patients admitted with acute lover failure (ALF) or acute liver injury (ALI). The study closed enrollment in August 2019 after enrolling 3,364 patients with ALF or ALI. The entire study conformed to the [2013 Declaration of Helsinki](https://www.wma.net/policies-post/wma-declaration-of-helsinki-ethical-principles-for-medical-research-involving-human-subjects/) and the [2018 Declaration of Istanbul](https://www.declarationofistanbul.org/the-declaration). All 23 clinical sites were in compliance with the requirements of their local institutional IRB. A certificate of confidentiality was obtained from the National Institutes for Mental Health for the entire study.

**Hepatitis B Research Network (HBRN) Cohort**

The Hepatitis B Research Network (HBRN) provided detailed data and serum samples from carefully adjudicated and prospectively enrolled patients with classical acute hepatitis B (AH) that presented without evidence of hepatic failure, as a comparison group. HBRN is a consortium of investigators from 21 geographically diverse clinical centers across the United States and Canada also funded by NIDDK, registered on ClinicalTrials.gov (NCT01263587). A prospective longitudinal study of patients diagnosed with chronic HBV infection was the primary aim of the Network [1, 2]. As an ancillary study to the main registry, 60 patients diagnosed at study sites with acute hepatitis B were enrolled and followed for up to at least 12 months to determine outcomes [3]. The initial analysis sample of 60 acute hepattis B participants were adjudicated by the HBRN diagnostic review committee as follows: positive IgM anti-HBc detected with or without HBsAg within 30 days of onset of illness; clinical picture consistent with acute hepattis B; absence of evidence of pre-existing HBV infection with reactivation; confirmation of the diagnosis in the treating physician narrative. Patients were deemed to have HBV reactivation if previously known to be HBV infected, had acute elevation of ALT and HBV DNA, with negative or very low-titer IgM anti-HBc. Those with apparent HBV reactivation, antibodies to hepatitis C virus and hepatitis delta virus were excluded. Patients deemed to have acute hepatitis B were generally outpatients or had brief hospitalizations and were enrolled at baseline, weeks 1, 2, 4, 6, 8, 12, 18, 24, and 48, with flexibility to alter the schedule depending on resolution of the illness. Patients were encouraged to remain in the study for at least 24-weeks to document full resolution and viral clearance. For the present study, 13 of the 60 patients with acute hepatitis B were selected randomly, if sufficient serum samples and complete data were available.

**Quantitative HBsAg testing**

Serial dilutions of samples within the linear range were compared to a standard curve calibrated to the HBsAg genotype A international reference standard (NIBSC Code# 03/262). Quantities were expressed as log IU per mL.

**HDV RNA**

Serum RNA was extracted using QIAamp viral RNA mini kit (QIAGEN, catalog no. 52904) from approximately 140 to 200 µL of serum. Serum HDV RNA was tested by real-time quantitative PCR using a one-step RT-PCR protocol with TaqMan RNA-to-CT One-Step Kit chemistry, random hexamers, and primers with an MGB-probe spanning nucleotides 894 to 971, as described previously [4].

**HBV DNA**

Serum DNA was extracted from approximately 200 µL of serum or plasma. Real-time quantitative PCR was performed using TaqMan chemistry with cycling conditions based on the manufacturer's (ABI) recommendations. Negative and no-template controls were included in all test runs. Serum HBV DNA was quantified using methods described previously [5]. Briefly, extracted total DNA was analyzed using TaqMan™ Fast Advanced Master Mix (ThermoFisher) and primers with an MGB-probe targeting near the 5’ end of the S gene. The assay incorporated standards spanning a 6-log dynamic range, with a lower limit of detection of approximately 50-200 copies.

**HBV genotyping**

HBV genotyping was performed using a commercially available line-probe assay (INNO-LiPA HBV Genotyping Assay; Fujirebio, catalog no. 80070). Viral DNA was extracted from 100 µL of serum using a commercially available kit according to the manufacturer’s instructions (DNeasy Blood and Tissue Kit, QIAGEN, catalog no. 69504). Nested PCR amplification was conducted following the manufacturer’s instructions using Taq 2× Master Mix (New England Biolabs, catalog no. M0270L) to generate a biotinylated amplicon for downstream hybridization. The outer PCR primers amplified a 409-bp region of the HBV polymerase gene encompassing domains B and C. The second-round PCR used biotinylated nested primers to amplify a 342-bp fragment. Following amplification, the biotinylated PCR products derived from the HBsAg open reading frame were hybridized to genotype-specific oligonucleotide probes immobilized as parallel lines on membrane-based strips. Genotypes were assigned according to the manufacturer’s interpretation guidelines: when multiple genotype-specific bands corresponding to one genotype were present along with a single band from another genotype, only the genotype with multiple reactive bands was reported. The sole exception to this rule was genotype G, which is identified exclusively by reactivity of the final line on the strip.

**Next-Generation Sequencing of HBV, Genome Assembly, and Sequence Analysis**

Libraries were prepared using 1000 ng of sample using the Adapter-Barcoded workflow of the Preparing multiplexed amplicon libraries using SMRTbell® prep kit 3.0’ protocol (Pacific Biosciences). Libraries were pooled in an equimolar ratio and the size and purity of the pooled library were assessed using a Bioanalyzer (Agilent). The pooled libraries were run on 8M SMRTCells using version 2.0 sequencing reagents on a Sequel IIe sequencer (Pacific Biosciences) with Instrument Control Software Version 11.0.0.144466, a 0.5-hour pre-extension and 10-hour movie collection time per SMRT Cell. Circular consensus sequence (CCS/HiFi) reads were generated off-instrument from the initial subread data using the pb_ccs workflow and demultiplexed using the pb_demux_ccs workflow within PacBio SMRTLink version 11.0.0.146107.

Demultiplexed reads in BAM format were filtered by length (minimum 825 bp and maximum 1300 bp) and read quality, using a quality score threshold of rq:f ≥ 0.99. Filtered BAM files were converted to FASTQ format using bam2fastq from the SMRT Link Tools software package (version 10.1). Reads were subsequently subsampled to a depth of 5000× coverage to reduce computational load and standardize analysis across samples. The subsampling process was performed in triplicate to ensure reproducibility. To generate an initial assembly for genotype determination, subsampled reads were aligned to the HBV reference genome (GenBank accession number X02763.1) using the pbmm2 wrapper for minimap2 (SMRT Link Tools version 10.1). Consensus genomes were generated using Pilon (version 1.24) [6]. Sample genotypes were determined using the Genome Detective Hepatitis B Virus Phylogenetic Typing Tool, available at <https://www.genomedetective.com/app/typingtool/hbv/>.

Following genotype assignment, subsampled reads were aligned to the corresponding genotype-specific reference genome using the minimap2 wrapper pbmm2 (SMRT Link Tools version 10.1). Reference sequences used for each genotype were as follows: AB116077.1 for genotype A; D00329.1 for genotype B; AY123041.1 for genotype C; AB033559.1 for genotype D; X75664.1 for genotype E; KX264500.1 for genotype G; and KX264501.1 for genotype H. Variant calling and generation of consensus sequences were performed using Pilon.

Consensus genomes were further polished by iterative mapping of the FASTQ reads back to the consensus sequence for each sample until no additional single-nucleotide polymorphisms (SNPs) or insertions/deletions (indels) were detected. To identify low-frequency variants within each sample, reads were mapped to the assembled genome sequence from the patient's first available visit, and intrahost variants were called using **iVar**, a tool designed for low-frequency variant detection [7].

#### **Phylogenetic Analysis**

Multiple sequence alignment of consensus genomes was performed using MAFFT version 7.490 [8] within the Geneious software platform (Geneious Ltd., <https://www.geneious.com/>). A maximum-likelihood phylogenetic tree was inferred using IQ-TREE version 2.2.2.6 [9] using the GTR+F+G4 substitution model. Statistical support for the inferred tree topology was assessed using 1,000 ultrafast bootstrap replicates.

**Highly Multiplexed CodePlex Chip-Based Serum Proteomic Analysis**

Cryopreserved serial serum samples from patients with hepatitis B were thawed at room temperature and mixed thoroughly by pipetting prior to loading. An aliquot of 5.5 µL from each sample was dispensed into individual macrochambers of a CodePlex chip pre-patterned with a complete 19-plex antibody array. A 2% BSA/PBS solution was used as background control. The chip was loaded into an IsoLight automation system, and proteins levels were measured by fluorescence-based ELISA. Data were analyzed using IsoSpeak software with the IsoPlexis Human Innate Immune Panel, which measures the following analites: Granzyme B, IFN-γ, MIP-1α, TNF-α, GM-CSF, IL-7, IL-8, IL-15, IP-10, MIP-1β, IL-4, IL-10, sCD137, IL-1β, IL-6, MCP-1, EGF, PDGF-BB, VEGF. Protein concentrations were calculated using standard curves generated for each CodePlex chip lot.

**REFERENCES**

1. Di Bisceglie AM, King WC, Lisker-Melman M, Khalili M, Belle SH, Feld JJ, Ghany MG, et al. Age, race and viral genotype are associated with the prevalence of hepatitis B e antigen in children and adults with chronic hepatitis B. J Viral Hepat 2019;26:856-865.

2. Ghany MG, Perrillo R, Li R, Belle SH, Janssen HL, Terrault NA, Shuhart MC, et al. Characteristics of adults in the hepatitis B research network in North America reflect their country of origin and hepatitis B virus genotype. Clin Gastroenterol Hepatol 2015;13:183-192.

3. Sterling RK, Wahed AS, Cloherty G, Hoofnagle JH, Lee WM, Hepatitis BRNI. Acute Hepatitis B Virus Infection in North American Adults. Clin Gastroenterol Hepatol 2023;21:1881-1892 e1884.

4. Engle RE, De Battista D, Danoff EJ, Nguyen H, Chen Z, Lusso P, Purcell RH, Farci P. Distinct Cytokine Profiles Correlate with Disease Severity and Outcome in Longitudinal Studies of Acute Hepatitis B Virus and Hepatitis D Virus Infection in Chimpanzees. mBio 2020;11.

5. Engle RE, Bukh J, Alter HJ, Emerson SU, Trenbeath JL, Nguyen HT, Brockington A, et al. Transfusion-associated hepatitis before the screening of blood for hepatitis risk factors. Transfusion 2014;54:2833-2841.

6. Walker BJ, Abeel T, Shea T, Priest M, Abouelliel A, Sakthikumar S, Cuomo CA, et al. Pilon: an integrated tool for comprehensive microbial variant detection and genome assembly improvement. PLoS One 2014;9:e112963.

7. Grubaugh ND, Gangavarapu K, Quick J, Matteson NL, De Jesus JG, Main BJ, Tan AL, et al. An amplicon-based sequencing framework for accurately measuring intrahost virus diversity using PrimalSeq and iVar. Genome Biol 2019;20:8.

8. Katoh K, Misawa K, Kuma K, Miyata T. MAFFT: a novel method for rapid multiple sequence alignment based on fast Fourier transform. Nucleic Acids Res 2002;30:3059-3066.

9. Nguyen LT, Schmidt HA, von Haeseler A, Minh BQ. IQ-TREE: a fast and effective stochastic algorithm for estimating maximum-likelihood phylogenies. Mol Biol Evol 2015;32:268-274.

**Supplementary Table 1**. Basal Core Promoter and Precore Variants in HBV Genotypes Other Than A and D

| **Patient** | **Phenotype** | **HBV genotype** | **Variants** | | |
| --- | --- | --- | --- | --- | --- |
|  |  |  | **A1762T** | **G1764A** | **G1896A** |
| 3693 | Acute liver failure | E | - | - | - |
| 3961 | Acute liver failure | C | + | + | + |
| 4947 | Subacute liver failure | C | + | + | - |
| 220118MGF | Classic acute hepatitis | B | - | - | + |
| 4413 | ALF on CHB | C | + | + | - |
| 4510 | ALF on CHB | G | + | + | + |
| 4534 | ALF on CHB | B | - | - | + |
| 4655 | ALF on CHB | B | - | - | + |
| 4860 | ALF on CHB | C | - | - | - |
| 6282 | ALF on CHB | H | - | - | - |

ALF denotes acute liver failure; CHB denotes chronic hepatitis B.

**Supplementary Table 2**. Summary of modified generalized estimating equations (GEE) coefficient values by phenotype and biomarker

| **Characteristic** | **Classic AH**  **(n = 13)** | **ALI**  **(n = 7)** | **Acute Liver Failure**  **(n = 7)** | **Subacute Liver Failure**  **(n = 11)** | **ALF on CHB**  **(n = 8)** |
| --- | --- | --- | --- | --- | --- |
| ***Clinical features*** |  |  |  |  |  |
| ALT (IU/mL) | -0.812 [<.001] | -0.074 [0.081] | -0.072 [0.025] | -0.052 [0.024] | -0.08 [<.001] |
| AST (IU/mL) | -0.704 [<.001] | -0.15 [0.005] | -0.124 [0.022] | -0.058 [0.004] | -0.127 [0.004] |
| Bilirubin (mg/dL) | -0.331 [<.001] | -0.033 [0.068] | 0.016 [0.481] | 0.008 [0.188] | 0.007 [0.536] |
| INR | -0.011 [0.057] | -0.013 [0.214] | -0.015 [0.250] | 0.002 [0.829] | 0.008 [0.328] |
| ***Virologic features*** |  |  |  |  |  |
| HBsAg (IU/mL) | -2.047 [<.001] | -0.162 [0.371] | -0.136 [0.107] | -0.079 [0.097] | -0.021 [0.690] |
| IgM anti-HBc (IU/mL) | -0.759 [<.001] | -0.043 [0.623] | -0.035 [0.379] | -0.041 [0.666] | 0 [0.997] |
| HBV DNA (IU/mL) | -1.907 [<.001] | -0.144 [0.309] | -0.206 [0.030] | -0.038 [0.330] | -0.149 [0.323] |
| HBcrAg (U/mL) | -1.241 [<.001] | -0.076 [0.448] | -0.273 [0.046] | -0.117 [0.081] | -0.103 [0.319] |
| HBV RNA (U/mL) | N/A | 0.124 [0.250] | -0.25 [0.106] | -0.058 [0.333] | -0.006 [0.919] |
| ***Type 1 immunity*** |  |  |  |  |  |
| Granzyme B (pg/mL) | -0.145 [0.275] | 0.029 [0.842] | -0.231 [0.022] | 0.099 [0.229] | 0.114 [0.095] |
| IFN-γ (pg/mL) | -0.404 [0.002] | 0.113 [0.046] | -0.094 [0.169] | 0.07 [0.116] | 0.009 [0.734] |
| IL-1β (pg/mL) | 0.067 [0.298] | -0.017 [0.364] | -0.046 [0.348] | 0.011 [0.216] | 0.009 [0.749] |
| IL-8 (pg/mL) | -0.057 [0.406] | 0.043 [0.697] | -0.048 [0.388] | 0.027 [0.479] | 0.025 [0.733] |
| IL-15 (pg/mL) | N/A | 0.04 [0.311] | 0.004 [0.642] | 0.019 [0.193] | -0.043 [0.333] |
| IP-10 (pg/mL) | -0.818 [<.001] | -0.03 [0.531] | -0.043 [0.308] | -0.061 [0.024] | 0.002 [0.944] |
| MIP-1α (pg/mL) | -0.058 [0.652] | -0.072 [0.575] | -0.155 [0.038] | -0.022 [0.736] | 0.11 [0.110] |
| MIP-1β (pg/mL) | -0.218 [<.001] | -0.08 [0.090] | 0.002 [0.930] | -0.02 [0.146] | -0.01 [0.651] |
| TNF-α (pg/mL) | 0.101 [0.354] | 0.052 [0.550] | -0.124 [0.058] | 0.047 [0.027] | 0.107 [0.081] |
| ***Type 2 immunity*** |  |  |  |  |  |
| IL-4 (pg/mL) | 0.108 [0.546] | 0.105 [0.274] | -0.093 [0.020] | 0.082 [0.137] | 0.129 [0.120] |
| IL-6 (pg/mL) | -0.165 [0.184] | 0.075 [0.155] | 0.009 [0.881] | 0.067 [0.299] | 0.063 [0.098] |
| IL-7 (pg/mL) | -0.566 [0.024] | 0.122 [0.157] | -0.137 [0.050] | 0.055 [0.464] | 0.089 [0.255] |
| IL-10 (pg/mL) | -0.101 [0.410] | -0.002 [0.970] | -0.058 [0.245] | 0.005 [0.882] | -0.039 [0.500] |
| MCP-1 (pg/mL) | 0.074 [0.601] | -0.022 [0.730] | -0.022 [0.479] | -0.008 [0.710] | 0.026 [0.357] |
| sCD137 (pg/mL) | -0.017 [0.916] | 0.129 [0.092] | -0.126 [0.037] | 0.095 [0.226] | 0.094 [0.193] |
| ***Growth factors*** |  |  |  |  |  |
| EGF (pg/mL) | 0.025 [0.771] | -0.123 [0.174] | -0.102 [0.294] | -0.066 [0.061] | -0.084 [0.364] |
| GM-CSF (pg/mL) | 0.01 [0.892] | -0.007 [0.365] | -0.044 [0.230] | 0.003 [0.719] | -0.01 [0.822] |
| PDGF-BB (pg/mL) | -0.027 [0.413] | -0.039 [0.430] | -0.045 [0.204] | -0.045 [0.120] | -0.075 [0.047] |
| VEGF (pg/mL) | -0.293 [0.116] | -0.135 [0.224] | 0.075 [0.348] | 0.052 [0.332] | -0.099 [0.131] |

Data are shown as modified GEE coefficient [*p* value]. ALI denotes acute liver injury; ALF denotes acute liver failure; CHB denotes chronic hepatitis B.

**Supplementary Figure 1**. Cytokine profiles across five clinical phenotypes at hospital admission. (A) Serum levels of type 1 immunity-related cytokines and (B) type 2 immunity-related cytokines, and (C) growth factors in patients with classic acute hepatitis (AH) (green), ALI (orange), acute liver failure (red), subacute liver failure (yellow), and acute liver failure on CHB (blue). Box- and-whisker plots represent the median (horizontal line), interquartile range (25^th^ and 75^th^ percentiles; bottom and top of each box) and the lower and upper whiskers, which cover values within 1.5 × IQR of the first and third quartiles, respectively. ALF denotes acute liver failure. CHB, denotes chronic hepatitis B. Statistical analysis using the Kruskal–Wallis test followed by Dunn’s multiple-comparison test showed no statistically significant differences among the groups.

**Supplementary Figure 2**. Cytokine profiles among the three forms of acute liver failure at admission. (A) Serum levels of type 1 immunity-related cytokines, and (B) type 2 immunity–related cytokines, and (C) growth factors in patients with acute liver failure (red), subacute liver failure (yellow), and ALF on CHB (blue). Box- and-whisker plots represent the median (horizontal line), interquartile range (25^th^ and 75^th^ percentiles; bottom and top of each box) and the lower and upper whiskers, which cover values within 1.5 × IQR of the first and third quartiles, respectively. Statistical analysis using the Kruskal–Wallis test followed by Dunn’s multiple-comparison test showed no statistically significant differences among the groups.

**Supplementary Figure 3**. Longitudinal changes in clinical, virological and cytokine levels overtime in patients with classic acute hepatitis (AH), acute liver injury (ALI), acute liver failure, subacute liver failure, and acute liver falure on chronic hepatitis B (CHB). Serum levels of each parameter were quantified daily in all groups, except in patients with classic AH, for whom samples were collected at hospital admission, twoo weeks later, and subsequently at 6 months. **The pink shaded areas denote significant changes during longitudinal analysis, whereas the grey areas denote no significant changes over time; *p* values are shown in Supplementary Table 2.**
